# Reply to Mazzei et al. Some Concerns from a Radiological Point of View. Comment on “Huang et al. Outcomes of Conversion Surgery for Metastatic Gastric Cancer Compared with In-Front Surgery plus Palliative Chemotherapy or In-Front Surgery Alone. *J. Pers. Med.* 2022, *12*, 555”

**DOI:** 10.3390/jpm12071069

**Published:** 2022-06-30

**Authors:** Hao-Wei Kou, Jun-Te Hsu

**Affiliations:** Department of General Surgery, Chang Gung Memorial Hospital at Linkou, College of Medicine, Chang Gung University, Taoyuan 33305, Taiwan; b9602039@cgmh.org.tw

We thank the authors for their interest in our article “Outcomes of Conversion Surgery for Metastatic Gastric Cancer Compared with In-Front Surgery Plus Palliative Chemotherapy or In-Front Surgery Alone” [1]. We also acknowledge their critical insight and sharing their data regarding the evaluation on peritoneal carcinomatosis [2].

Patients with stage IV gastric cancer are a heterogenous population with various disease characteristics and extension. One of the crucial points for conversion surgery is to select the right patients who are feasible and may benefit from this therapeutic approach. Therefore, it is important to assess treatment responses accurately to tailor the following treatment plans. The authors proposed their opinion of applying combined peritoneal assessment based on computed tomography and RECIST 1.1 criteria to evaluate tumor response after therapies, especially for peritoneal carcinomatosis. Indeed, computed tomography had limitations on determining the peritoneal metastasis, particularly for those low-volume tumors on peritoneal surfaces or at a difficult location [3]. Nonetheless, multidetector computed tomography is the most widely used tool for detection and evaluation of peritoneal carcinomatosis [4], which is still the first choice modality for assessing peritoneal conditions in gastric cancer, suggested by the ESCO guidelines [5]. Except for computed tomography scans, other diagnostic modalities, including magnetic resonance images, positron emission tomography scans, gastrointestinal endoscopies, or a biopsy of suspicious lesion, could be adopted to appraise treatment response [6]. In addition, clinical data, such as changes of patients’ general performance status, tumor markers, body weight and nutrition status, as well as laboratory examinations, can also provide valuable information to submit the patient into a gastric cancer team for the discussion of conversion surgery. Due to the drawbacks of noninvasive image modalities, diagnostic laparoscopy remains a useful method to clarify peritoneal carcinomatosis with the highest accuracy [7,8]. Furthermore, cytoreductive surgery (CC0 or CC1) plus hyperthermic intraperitoneal chemotherapy might offer an alternative to improve the overall survival for fit patients with a peritoneal cancer index score < 12 [9]. However, a large-scale randomized trial is needed to validate this approach. More efforts should also be made to overcome the obstacle of detection of peritoneal metastasis.
